# Brown Sequard syndrome in a patient with Klippel-Feil syndrome following minor trauma: a case report and literature review

**DOI:** 10.1186/s12891-023-06760-9

**Published:** 2023-09-11

**Authors:** Shuyi Zhang, Zhao Wang, Shuao Zhang, Chenshui Lu, Zhengpeng Liu, Chan Kang, Fengfei Lin, Dongze Lin, Licai Huang, Yilong Zhang

**Affiliations:** 1 Department of Orthopedics, Fuzhou second Hospital, Fuzhou, 350100 Fujian China; 2 Department of Spine Surgery, Affiliated Hospital of Chengde Medical College, Chengde, 067000 Hebei China; 3 Department of Orthopedics, Chungnam National University Hospital, Daejeon, 35015 Republic of Korea; 4 School of Civil Engineering, Fujian University of Technology, Fuzhou, 350000 Fujian China; 5 Department of Foreign Languages, Fu Zhou University, Fuzhou, 350100 Fujian China

**Keywords:** Klippel-Feil syndrome(KFS), Brown-Sequard syndrome(BSS), Spinal cord injury (SCI), ASIA (American Spinal Injury Association) Impairment Scale, Cervical

## Abstract

**Background:**

There are some cases of Klippel-Feil syndrome with spinal cord injury in clinical work. However, there is no literature report on Brown-Sequard syndrome after trauma. We report a case of Brown-Sequard syndrome following minor trauma in a patient with KFS type III. Her Brown-Sequard syndrome is caused by Klippel-Feil syndrome.

**Case presentation:**

We found a 38-year-old female patient with KFS in our clinical work. She was unconscious on the spot following a minor traumatic episode. After treatment, her whole body was numb and limb activity was limited. Half an hour later, she felt numb and weak in the right limb and weak in the left limb. She had no previous hypertension, diabetes, or coronary heart disease. After one-month treatment of medication, hyperbaric oxygen, rehabilitation, and acupuncture in our hospital, her muscle strength partially recovered, but the treatment effect was still not satisfactory. Then, she underwent surgical treatment and postoperative comprehensive treatment, and rehabilitation training. She was able to take care of herself with assistance, and her condition improved from grade B to grade D according to the ASIA (ASIA Impairment Scale) classification.

**Conclusion:**

KFS, also known as short neck deformity, is a kind of congenital deformity characterized by impaired formation and faulty segmentation of the cervical spine, often associated with abnormalities of other organs. The cervical deformity in patients with KFS can alter the overall mechanical activity of the spine, as well as the compensatory properties of the spine for decelerating and rotatory forces, thus increasing the chance of spinal cord injury (SCI) following trauma. Many mechanisms can make patients more susceptible to injury. Increased range of motion of the segment adjacent to the fused vertebral body may lead to slippage of the adjacent vertebral body and altered disc stress, as well as cervical instability. SCI can result in complete or incomplete impairment of motor, sensory and autonomic nervous functions below the level of lesion. This woman presented with symptoms of BSS, a rare neurological disorder with incomplete SCI. Judging from the woman’s symptoms, we concluded that previously she had KFS, which resulted in SCI without fracture and dislocation following minor trauma, with partial BSS.

After the comprehensive treatment of surgery, hyperbaric oxygen, rehabilitation therapy, and neurotrophic drugs, two years later, we found her symptoms significantly improved, with ASIA Impairment Scale from grade B to grade D, and her ability to perform activities of daily living with aids.

## Background

Klippel-Feil syndrome (KFS) was first identified in 1912 by Maurice Klippel and Andre Feil, who reported a patient with multiple fused cervical vertebrae, a congenital spinal deformity that clinically often presents with multiple abnormal fusions of the cervical vertebrae and congenital spinal stenosis, along with a short neck, limited cervical mobility, or low occipital hairline [1], Intervertebral fusion may also occur in a single segment [2]. Most cases of KFS are sporadic and it is generally accepted that KFS is caused by several chromosomal abnormalities, including autosomal dominant inheritance: the autosomal GDF6 gene on autosome 8 [3], and the autosomal GDF3 gene on autosome 12 [4]; autosomal stealth inheritance: MEOX1 on chromosome 17 [5], and also other Several loci Hox, SGM1, and PAX1 [6, 7]. All of these different genetic mutations manifest as KFS, a phenomenon known as genetic heterogeneity of KFS [2, 6, 8–10]. KFS occurs in approximately 1 in 40,000 births with a slight female predilection [11]. Also, KFS may be associated with developmental defects in many other organ systems, including the inner ear, spinal cord, heart, and genitourinary tract. We report a case of Brown-Sequard syndrome (BSS)following minor trauma in a patient with KFS type III.

## Case presentation

**Fig. 1:**
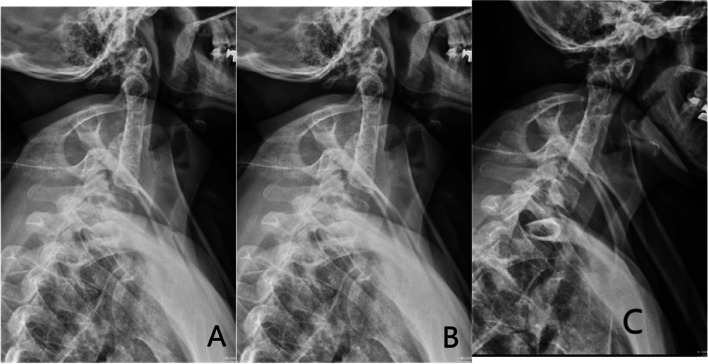
DR of Patient's pre-operative cervical spine (**A** neutral position, **B** posterior extension, **C** anterior flexion)

**Fig. 2:**
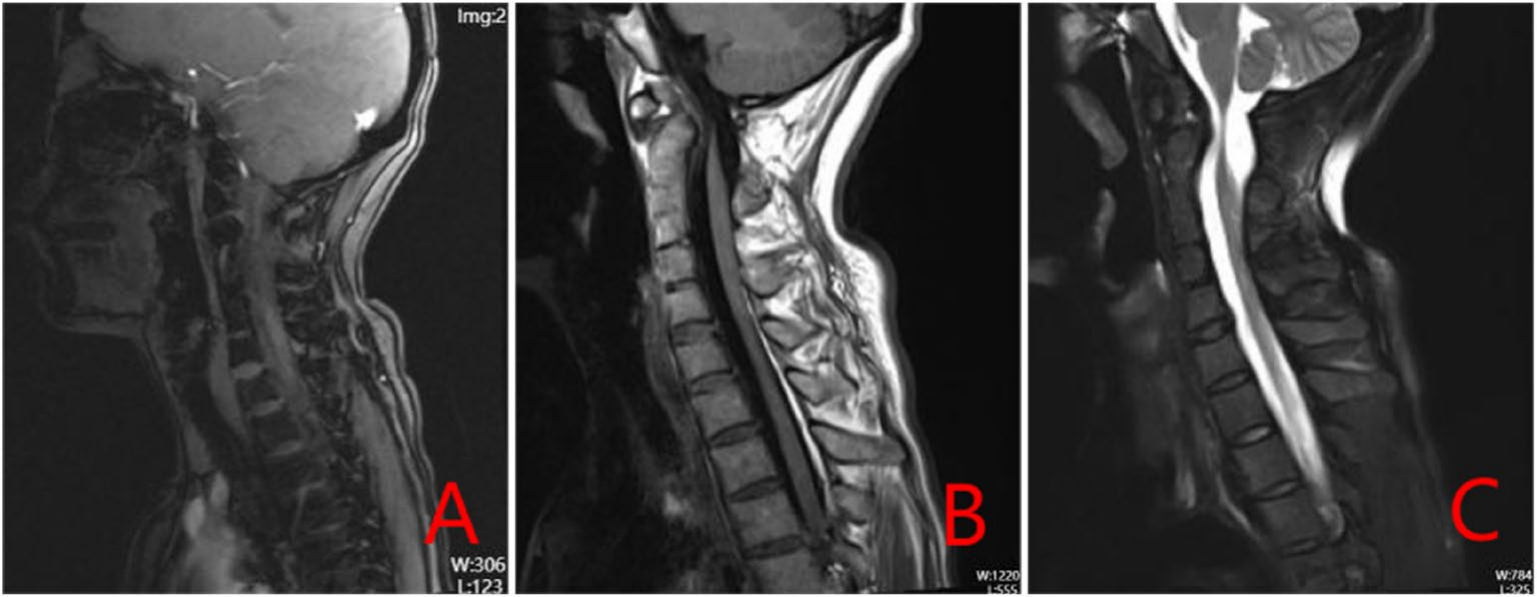
Patient's pre-operative sagittal MRI (**A** T1W1,**B** T2W1,**C** T2W1 STIR)

**Fig. 3:**
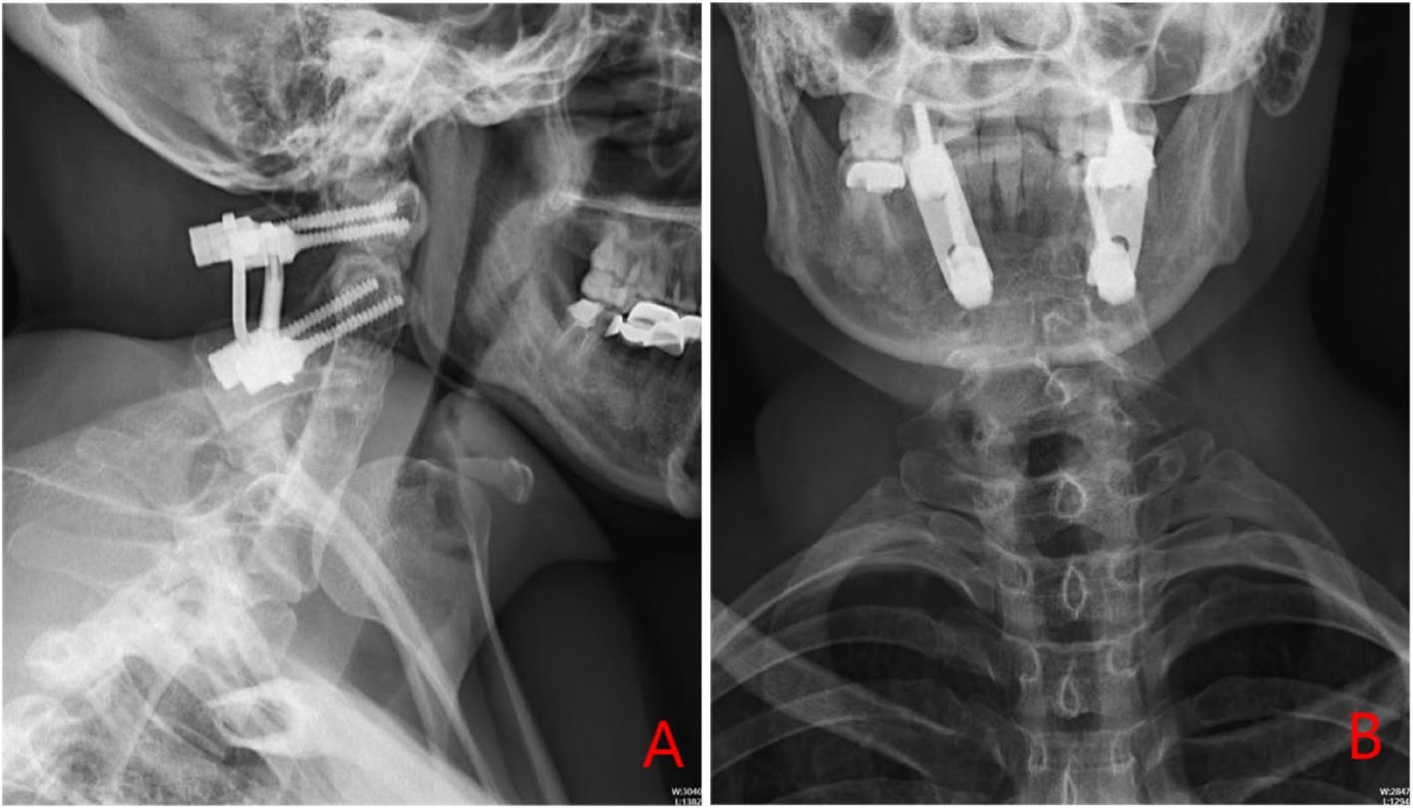
Patient's post-operative DR (**A** Sagittal position, **B** Coronal position)

A 38-year-old woman developed numbness in the right limb and weakness and limited movement in the left limb following a fall from hitting her head on a door beam. She was unconscious on the spot. After treatment, her whole body was numb and limb activity was limited. Half an hour later, she felt numb and weak in the right limb and weak in the left limb. She had no previous hypertension, diabetes, or coronary heart disease. 13 years ago, she developed numbness in her right hand after pregnancy and was diagnosed with congenital fusion of cervical C2-5, which was not treated at that time (Fig. 1). Her symptoms had improved and had not interfered with her normal life. There was no diplopia, slurred speech, hiccups, nausea and vomiting, dysphagia, urinary incontinence, and no corresponding symptoms such as facial sensory abnormalities. Physical examination revealed a short neck, limited cervical mobility, and low occipital hairline. Below the C3 level of spinal cord, bounded by the anterior median line, there were different sensory and motor abnormalities from left to right. The patient had decreased pinprick and temperature sensation on the right side and normal pinprick sensation on the left side. Her sense of spatial position was normal. There was increased muscle tone in the right upper and lower limbs and decreased muscle tone in the left upper and lower limbs. The muscle strength of the left upper and lower limbs was 0 out of 5 and the strength of the right upper and lower limbs was 4 out of 5. After conservative treatment, her muscle strength gradually recovered. 10 days later, some of the muscle strength showed changes, and the muscle strength of the key muscle groups was as follows: shrugging shoulder muscle strength (left 2, right4), elbow flexion muscle strength (left 2, right 4), elbow extension muscle strength (left 2, right 4), wrist flexion muscle strength (left 1, right 4). finger flexion muscle strength (left 1, right 4), finger extension muscle strength (left 1, right 3), hip flexion (left 2, right 4), knee extension (left 2, right 4), dorsalis pedis (left 3, right 4), plantarflexion (left 3, right 4), and hyperreflexia of the biceps and triceps tendons bilaterally. Abdominal wall reflexes were present, knee and Achilles tendon reflexes were hyperactive, patellar clonus was positive on the right, patellar clonus was positive on the left, ankle clonus was positive on the right and ankle clonus was positive on the left. The dorsalis pedis artery was palpable bilaterally. The bilateral Hoffman's sign was positive. Babinski's sign was positive and Kernig's sign was positive. The findings of Magnetic resonance imaging (MRI) (Fig. 2) in the neck revealed that small C2-5 vertebral body with partial fusion of the vertebral body; increased anterior atlantoaxial space, posterior superior displacement of the cardinal vertebrae, the narrowing of the spinal canal at the corresponding level and marked compression and thinning of the spinal cord (C1-2 joint instability, discontinuity of the odontoid process, congenital fusion of cervical C2-5). posterior protrusion of the C7-T1 intervertebral disc, with compression of the corresponding dural sac. No significant abnormal signs were seen in the cervical medulla. We considered that the woman sustained BBS because she had previously suffered from KFS, which according to ASIA(American Spinal Injury Association) Impairment Scale [12] was a grade B: incomplete injury. After admission, the woman was given methylcobalamin for neurotropism and tizanidine to reduce muscle tone and received acupuncture and hyperbaric oxygen therapy. After conservative treatment, her spinal cord oedema decreased and the numbness on the right side gradually subsided, but the results were still unsatisfactory so the doctor recommended surgery. She then underwent posterior decompression of the spinal canal, and lateral mass fixation between atlas and axis with screw-plate system (Fig. 3). After surgery, her numbness subsided and she continued to receive adenosine cobalamin for neurotropic treatment. She came to our hospital for a check 5 months later after the operation. The numbness of the right limb significantly decreased and the dysfunction of the limbs was slightly better than before. She could sit independently and stand with assistance, but she was still unable to take care of himself. She then underwent regular rehabilitation treatment in our hospital. 18 months later, the numbness of her limbs had disappeared and she was able to take care of herself with assistance, and her condition improved from grade B to grade D according to the ASIA classification.

## Discussion

KFS, also known as short neck deformity, is a kind of congenital deformity characterized by impaired formation and faulty segmentation of the cervical spine, often associated with abnormalities of other organs [1]. The clinical management of patients with KFS involves reduced risk of trauma, because even minor trauma may cause abnormal symptoms [13]. A, Agrawal et al. reported a case of KFS after minor trauma resulting in disc herniation and spinal cord injury [14], OʼDonnel MD [15] reported a case of central spinal cord syndrome in a young man with KFS following moderate trauma. In this paper, we report a case of BSS following minor trauma in a patient with type III KFS who presented with right-limb and right-sided, and numbness and reduced pinprick below the C3 level, normal right-sided muscle strength, and weakness of the left side of limbs with normal sensation. The woman’s cervical spine x-ray images showed signs of KFS at C2-5. After the fall, she underwent posterior decompression of the spinal canal and lateral mass fixation between atlas and axis with screw-plate system at our hospital. Her muscle strength gradually recovered and numbness subsided compared to the preoperative period, but she was still unable to perform daily activities. In the follow-up checks, the woman gradually regained muscle strength and was able to take care of herself with the help of assistive devices eighteen months later after the injury.

The cervical deformity in patients with KFS can alter the overall mechanical activity of the spine, as well as the compensatory properties of the spine for decelerating and rotatory forces, thus increasing the chance of spinal cord injury (SCI) following trauma [16–20]. Many mechanisms can make patients more susceptible to injury. Increased range of motion (ROM) of the segment adjacent to the fused vertebral body may lead to slippage of the adjacent vertebral body and altered disc stress, as well as cervical instability [16, 17, 20–23]. At the same time, the increased ROM will bring about laxity and damage to the proximal segment ligaments [24, 25]. The increased ROM will also result in an increased incidence of cervical disc herniation as well as SCI [17, 20, 22, 23]. What is more, spinal stenosis may also come with it. Therefore, patients with KFS may present with SCI following minor trauma [17, 26]. Treatments for KFS and SCI depend on the severity of the vertebral instability, presence or absence of disc herniation, and related neurological deficits [20, 27, 28]. In this case, with KFS at the C2-5 segment and spinal stenosis at the fused segment, the woman was vulnerable to SCI after a minor traumatic episode [29]. We considered giving internal fixation to C2-3 to reduce the spinal cord injury from the ROM of the C2-3 segment.

SCI can result in complete or incomplete impairment of motor, sensory and autonomic nervous functions below the level of lesion [30]. This woman presented with symptoms of BSS, a rare neurological disorder with incomplete SCI [31]. There are two types of BSS. one is a typical spinal cord hemisection with ipsilateral paralysis, loss of ipsilateral proprioception and vibratory sensation, and loss of contralateral nociceptive and temperature sensation [32]. The numbness in this syndrome was caused by disruption of the corticospinal tracts of the lower crossings so that the motor manifestations of the medulla oblongata were on the same side as the injury. Although BSS is an easily identifiable condition, its exact incidence rate is difficult to determine. Besides, BBS may account for 2–4% of all traumatic SCI. The majority of patients currently have atypical BSS, which clinically presents with incomplete injuries, ipsilateral weakness, and the contralateral loss of pinprick and temperature sensation, but with intact proprioception and vibratory sensation. This results from the compression of the spinal cord with preservation of the posterior spinal cord [33, 34]. Some patients also presented with Honor Syndrome, mainly because of their impaired sympathetic fibers in the ipsilateral cervical spinal cord [32].

Judging from the woman’s symptoms, we concluded that previously she had KFS, which resulted in SCI without fracture and dislocation following minor trauma, with partial BSS. This partial BSS may be caused by the injury to her left ventral and lateral corticospinal tracts and the spinal thalamus tract.

## Conclusion

In this case, the woman with KFS sustained BBS following a minor trauma event. The key to her treatment was a comprehensive pre-operative assessment of her physical condition, adequate pre-operative preparation, adequate intraoperative decompression, and post-operative rehabilitation and medication. She came to our hospital two year later for a post-operative check. We found her symptoms significantly improved, with ASIA Impairment Scale from grade B to grade D, and her ability to perform activities of daily living with aids. Patients with KFS should be well protected in their daily life to reduce the possibility of trauma. Our conclusion after follow-up is that relatively good surgical outcomes can be achieved if patients with KFS undergo surgery within a short period following trauma, and we hope there will be more follow-up literature.

## Data Availability

The datasets used and/or analyzed during the current study are available from the corresponding author on reasonable request. Readers can access the data and material supporting the conclusions of the study by contacting Shuyi Zhang at 915368073@qq.com.

## Abbreviations

KFS: Klippel-Feil syndrome
BSS: Brown-Sequard syndrome
MRI: Magnetic resonance imaging
SCI: Chance of spinal cord injury
ROM: Range of motion
ASIA: (American Spinal Injury Association) Impairment Scale
