# Correction: Brown Sequard syndrome in a patient with Klippel-Feil syndrome following minor trauma: a case report and literature review

**DOI:** 10.1186/s12891-024-07170-1

**Published:** 2024-01-11

**Authors:** Shuyi Zhang, Zhao Wang, Shuao Zhang, Chenshui Lu, Zhengpeng Liu, Chan Kang, Fengfei Lin, Dongze Lin, Licai Huang, Yilong Zhang

**Affiliations:** 1 Department of Orthopedics, Fuzhou second Hospital, Fuzhou, Fujian 350100 China; 2 Department of Spine Surgery, Affiliated Hospital of Chengde Medical College, Chengde, Hebei 067000 China; 3 Department of Orthopedics, Chungnam National University Hospital, Daejeon, 35015 Republic of Korea; 4 School of Civil Engineering, Fujian University of Technology, Fuzhou, Fujian 350000 China; 5 Department of Foreign Languages, Fu Zhou University, Fuzhou, Fujian 350100 China

**Correction:** ***BMC Musculoskelet Disord*** **24** **, 722 (2023)**

10.1186/s12891-023-06760-9

Following the publication of the original article [1], the authors requested to insert funding institution “Fujian Provincial Clinical Medical Research Center for First Aid and Rehabilitation in Orthopaedic Trauma (2020Y2014).”

The affiliation of author “Chan Kang” has been changed to “Department of Orthopedics, Chungnam National University Hospital, Daejeon 35015, Republic of Korea”.

The original article [1] has been updated.
